# Gas-Phase Organosilane Self-Assembled Monolayers on Indium Tin Oxide Electrodes: Effects of Terminal Groups and Deposition Temperature

**DOI:** 10.3390/ma19122529

**Published:** 2026-06-11

**Authors:** Batdelger Ankhnybayar, Sang-Geon Park

**Affiliations:** 1 Department of Culture and Convergence Technology, Changwon National University, Changwon-si 51140, Gyeongsangnam-do, Republic of Korea; 2 Department of Mechatronics Convergence, Changwon National University, Changwon-si 51140, Gyeongsangnam-do, Republic of Korea

**Keywords:** OLED, self-assembled monolayer, gas-phase deposition, hole injection, surface engineering

## Abstract

**Highlights:**

- Gas-phase F_3_SAM deposition raised ITO work function and delivered eleven-fold higher luminance than bare ITO.
- CH_3_SAM improved performance at 10 min but multilayer roughening degraded devices at longer deposition times.
- NH_2_SAM lowered the work function and suppressed hole injection, increasing turn-on voltage progressively with deposition time.

**Abstract:**

Gas-phase thermal deposition was used to form three organosilane self-assembled monolayers on indium tin oxide (ITO) anodes: amine-terminated (NH_2_SAM), methyl-terminated (CH_3_SAM), and trifluoropropyl-terminated (F_3_SAM). Surface characterisation using water contact angle goniometry, ultraviolet photoelectron spectroscopy, and atomic force microscopy was combined with green organic light emitting diode (OLED) fabrication and J–V–L measurements to determine how terminal group chemistry and deposition time affect device performance. F_3_SAM increased the ITO work function from 3.72 to 4.47–4.72 eV, resulting in ultrasmooth surfaces (R_a_ = 0.20 nm) and a maximum luminescence of ~6000 cd m^−2^, eleven times higher than bare ITO (540 cd m^−2^). CH_3_SAM enhanced luminescence through surface passivation at 10 min (3374 cd m^−2^), but decreased quickly with extended deposition durations due to multilayer roughening. NH_2_SAM reduced the work function to 3.42 eV and gradually decreased hole injection, resulting in a turn-on voltage of 10–11 V after 180 min. These results indicate that terminal group polarity and deposition duration are the two most important parameters in gas-phase SAM engineering of ITO anodes for OLEDs.

## 1. Introduction

Since Tang and VanSlyke reported the first effective thin-film device in 1987 [1], organic light-emitting diodes have advanced significantly. Advances in materials and device engineering have brought OLEDs into commercial displays and lighting products, but improving charge injection efficiency at the anode interface is still an active research area, especially as device architectures push for lower operating voltages and longer operational lifetimes [2,3].

Most bottom-emission OLEDs use indium tin oxide as the transparent anode. Its optical transmittance surpasses 85% across the visible spectrum, and its work function ranges between 4.5 and 5.0 eV, depending on surface state and preparation process [4]. The concern is that this work function does not necessarily match the HOMO level of the surrounding hole-transport layer. NPB, one of the most extensively utilised HTL materials, has a HOMO of roughly 5.4 eV. Even a small energy offset at this interface raises the turn-on voltage, lowers current density at low bias, and ultimately restricts device luminance and efficiency [5,6].

Several techniques have been explored to resolve this mismatch, including UV-ozone treatment, oxygen plasma exposure, and the use of conductive polymer interlayers such PEDOT:PSS [7,8,9]. Self-assembled monolayers of organosilane coupling agents are gaining popularity as an alternative because they create covalently bound, monomolecular films on the ITO surface without appreciably altering optical transparency. More importantly, the work function of the modified surface can be tuned systematically by selecting the silane’s terminal functional group: electron-withdrawing groups increase the work function by creating a surface dipole that shifts the vacuum level upward, whereas electron-donating groups decrease it [10,11]. This level of control is difficult to attain with plasma treatments or polymer interlayers.

Most investigations on silane SAMs for OLED applications have employed solution-phase deposition, in which substrates are immersed in dilute silane solutions. This approach works, but it introduces complications: solvent impurities can cause uncontrolled silane polymerisation, rigorous anhydrous conditions are required, and a wet-chemistry phase is difficult to include into vacuum-based OLED manufacturing. Gas-phase deposition completely eliminates these difficulties. It is possible to generate uniform monolayers without using solvents or disrupting the dry processing environment required for device fabrication by vaporising the silane precursor and reacting it with a heated substrate inside a sealed vessel [12,13].

Three silane SAMs with different terminal groups, amine (NH_2_SAM), methyl (CH_3_SAM), and trifluoropropyl (F_3_SAM), were coated on ITO using gas-phase thermal deposition at 160 °C for 10, 60, and 180 min. Water contact angle measurements, UPS, and AFM were used to characterise the changed surfaces, and green-emitting OLEDs were constructed and tested to determine obvious linkages between surface chemistry, morphology, and device performance.

## 2. Materials and Methods

### 2.1. Materials

ITO-coated glass substrates with a 15 Ω sq^−1^ sheet resistance (AMG, Seoul, Republic of Korea) were utilised as received. Three silane coupling agents were chosen to represent chemically distinct terminal groups: 3-(trimethoxysilyl)propylamine (NH_2_SAM, ≥97%, Sigma-Aldrich, St. Louis, MO, USA), trimethoxy(propyl)silane (CH_3_SAM, >98%, Tokyo Chemical Industry, Japan), and trimethoxy(3,3,3-trifluoropropyl)silane (F_3_SAM, ≥95%), Gelest Inc., Morrisville, PA, USA). TCI Chemicals (Tokyo, Japan) supplied NPB and Alq_3_, while Alfa Aesar (Ward Hill, MA, USA) provided LiF and Al. All ingredients were used without any additional purification.

### 2.2. ITO Substrate Preparation

Cleaning involved a normal sequential ultrasonic bath operation in acetone, deionised water, and isopropyl alcohol for 5 min each, followed by nitrogen drying and baking at 160 °C for 5 min. Substrates were treated to UV-ozone for 10 min just before SAM deposition to eliminate surface organics and form a dense hydroxyl layer on the ITO surface.

### 2.3. SAM Deposition

Two deposition processes were employed to make SAM-modified ITO substrates. Both methods were used to measure contact angles for all three SAMs at deposition times of 10, 60, and 180 min. All following UPS, AFM, and OLED experiments were done on gas-phase deposited samples only. For gas-phase deposition, three neat silane droplets were deposited in a small PTFE boat within a larger sealed PTFE chamber. The ITO substrate was put next to the boat, and the chamber was heated in an oven at 160 °C for 10 min (A), 60 min (B), or 180 min. UV-ozone-treated ITO substrates were submerged in 1 M SAM solutions in ethanol for 10, 60, or 180 min at room temperature. Substrates were rinsed in an IPA ultrasonic bath, blown dry using nitrogen, then annealed at 120 °C for 10 min to solidify the monolayer. All gas-phase SAM-modified substrates were moved to the evaporation system within 30 min after preparation.

### 2.4. OLED Fabrication

Thermal evaporation was used to deposit organic and metal layers in a multi-source vacuum system (Terraleader, SGP lab, Changwon National University-Changwon (CWNU), Republic of Korea) with a base pressure of approximately 4 × 10^−6^ mbar. All layers were placed sequentially without breaking the vacuum. Green electroluminescence was produced by using NPB (50 nm) as the hole transport layer and Alq_3_ (50 nm) as both the emitting and electron transport layers. The electron injection layer and reflecting cathode were composed of LiF (0.6 nm) and Al (100 nm), respectively. Quartz crystal microbalances were used to detect the deposition rates at 1.5–2.0 Å s^−1^ for organic layers, 0.2–0.5 Å s^−1^ for LiF, and 5.5–6.0 Å s^−1^ for aluminium. Devices were described shortly after manufacturing under ambient circumstances.

### 2.5. Characterisation

Sessile-drop method was used to measure water contact angles using a CEO Phoenix 300 analyser (Surface Electro Optics, SGP lab, CWNU, Republic of Korea) and 2 µL droplets of deionised water. Five measurements were made from each sample and averaged. UPS was done using a He I source (hv = 21.22 eV) with a −5 V sample bias to resolve the secondary electron cutoff. The work function was recovered as WF = hv − E_SEC_ − 5 eV. Surface topography was characterised using tapping-mode AFM on a Park NX10 instrument (Park Systems, Central laboratory of CWNU, Republic of Korea). Images were examined for R_a_, R_q_, R_pv_, R_z_, R_sk_, and R_ku_ with XEI software 4.3.4 Build22. The J–V–L properties were recorded simultaneously utilising an McScience M3000 OLED test system, McSience Inc. (SGP lab, CWNU, Republic of Korea). 

## 3. Results

### 3.1. Water Contact Angle Analysis

**Figure 1:**
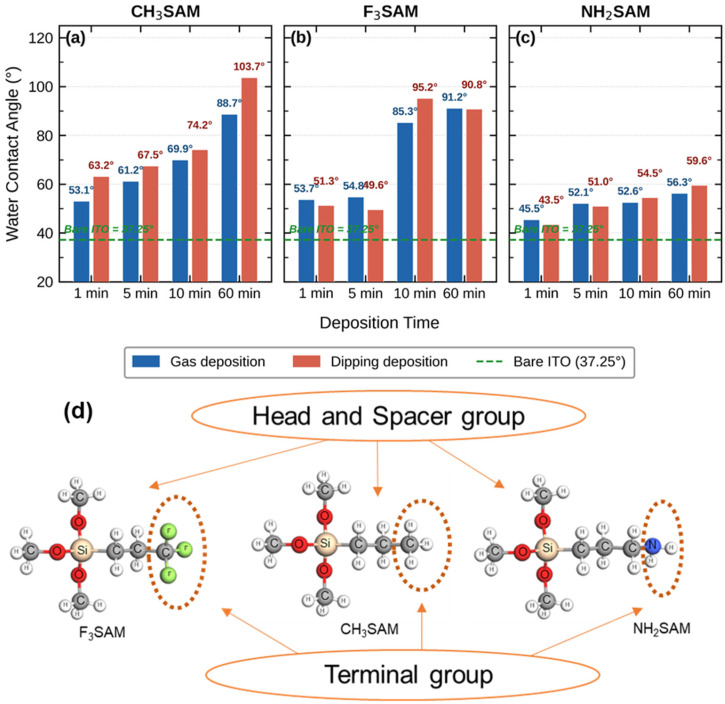
Water contact angle values of bare ITO and SAM-modified ITO surfaces deposited by gas-phase and dipping methods for 1, 5, 10, and 60 min (**a**–**c**), chemical structure of various SAMs (**d**).

The water contact angle (WCA) measurements are shown in Figure 1. The WCA of bare ITO was 37.25°, consistent with a hydrophilic surface dominated by hydroxyl groups following UV–ozone treatment. SAM deposition increased the WCA for all three silane types, confirming that organic functionalization occurred on the ITO surface. The degree of hydrophobicity depended strongly on the terminal group chemistry, with the general trend NH_2_SAM < F_3_SAM ≈ CH_3_SAM observed across all deposition conditions. CH_3_SAM exhibited a progressive increase in WCA from 53.08° at 1 min to 88.65° at 60 min for gas-phase deposition and from 63.20° to 103.72° for dipping deposition over the same range. The absence of a plateau in either scenario implies that surface coverage increased during the deposition window rather than approaching monolayer saturation [14]. Inter-silane condensation most likely led to multilayer development over time, as evidenced by the roughness measurements in Section 3.3. Dipping deposition yielded much higher WCA values than gas-phase, notably at 60 min, which is expected given the increased silane concentration available during liquid-phase immersion.

F_3_SAM surfaces achieved WCA values of 91.15° (gas) and 90.80° (dipping) after 60 min. F_3_SAM showed a sharp WCA jump between 5 and 10 min for both deposition procedures, unlike the other two SAMs. This kind of rapid transition often reflects the point at which discrete SAM islands on the surface unite into a continuous film—below that point, exposed ITO areas keep the total WCA low, while above it, the measurement reflects the fully covered surface. 

The early occurrence of this transition in F_3_SAM compared to CH_3_SAM indicates faster chemisorption kinetics for the trifluoropropylsilane precursor. Gas-phase and dipping procedures yielded roughly the same final WCA for F_3_SAM, indicating that both approaches provide equivalent surface coverage within 60 min for this silane.

NH_2_SAM resulted in the smallest WCA increases, ranging from 45.50° to 56.30° for gas-phase and 43.50° to 59.60° for dipping deposition. Even after 60 min, the surface remained somewhat wettable, which is understandable given that the amine end group preserves hydrogen bonding properties and does not completely suppress surface polarity in the same manner that fluorinated or methyl groups do. Gas and dipping results for NH_2_SAM were nearly identical, indicating that both techniques achieve a similar coverage ceiling under these conditions. 

Overall, the WCA results demonstrate successful silane attachment in all samples. The results also show that deposition kinetics and final coverage are not universal; they are dependent on both the terminal group and the deposition process, which has a direct impact on the surface attributes crucial to OLED performance [15].

### 3.2. Ultraviolet Photoelectron Spectroscopy

**Figure 2:**
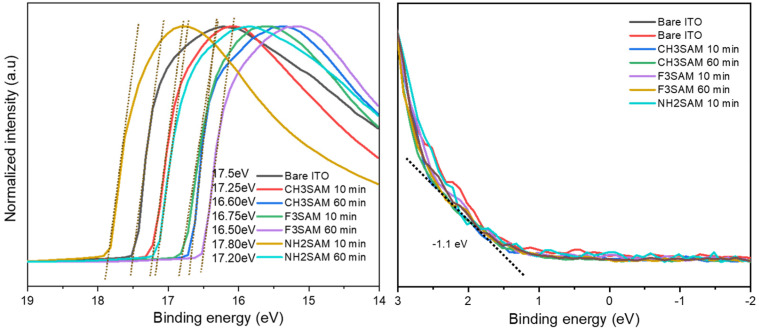
UPS He I secondary electron cutoff spectra of bare ITO and SAM-modified ITO surfaces at 10 and 60 min gas-phase deposition. Dashed lines indicate the cutoff positions used to extract the work function.

UPS measurements were performed to determine how each SAM influences the electrical structure of the ITO surface, specifically the work function (WF) and valence band on-set (HOMO/VB). The work function was calculated using the secondary electron cutoff (SEC) position: WF = hv − E_cutoff_, with hν = 21.22 eV (He I), as shown in Figure 2. Bare ITO has a work function of 3.72 eV and a valence band onset of 4.82 eV. SAM deposition affected these values in ways that were obviously dependent on the terminal functional group, and in most cases, the shifts grew more significant with longer deposition times.

After 10 min of NH_2_SAM, the work function of bare ITO was lowered to 3.42 eV, the lowest observed value across all samples. The amine-terminated SAM’s electron-donating -NH_2_ group forms a surface dipole, lowering the vacuum level at the ITO surface. At 60 min, the work function had largely recovered to 4.02 eV, probably because to a less coherently orientated monolayer at increased coverage, which partially balances out the dipole contribution. The low work function and low HOMO/VB position (4.52 and 5.12 eV) provide a greater energy offset compared to the NPB HOMO (~5.4 eV), explaining the elevated turn-on voltages found in NH_2_SAM devices.

Compared to bare ITO, CH_3_SAM enhanced the work function from 3.97 eV at 10 min to 4.62 eV at 60 min. The bigger shift at 60 min is most likely due to increased surface coverage rather than a substantial intrinsic dipole effect, as the methyl group has near-zero polarity. The related HOMO/VB values (5.07 and 5.72 eV) demonstrate a significant shift toward the NPB HOMO level for longer deposition times, but the device results indicate that the morphological degradation at 60 min (Section 3.3) cancels out any electrical benefit.

F_3_SAM achieved the highest work function values at both deposition times: 4.47 eV at 10 min and 4.72 eV at 60 min, with HOMO/VB locations of 5.57 and 5.82 eV. The increase is compatible with the electron-withdrawing behaviour of the -CF_3_ terminal group, which orients the surface dipole to raise the vacuum level. The HO-MO/VB of 5.57 eV for F_3_SAM at 10 min is close to the NPB HOMO level, reducing the hole injection barrier at the ITO/NPB interface. This is consistent with the much increased luminance seen for F_3_SAM-A devices [16].

### 3.3. AFM Surface Morphology

**Figure 3:**
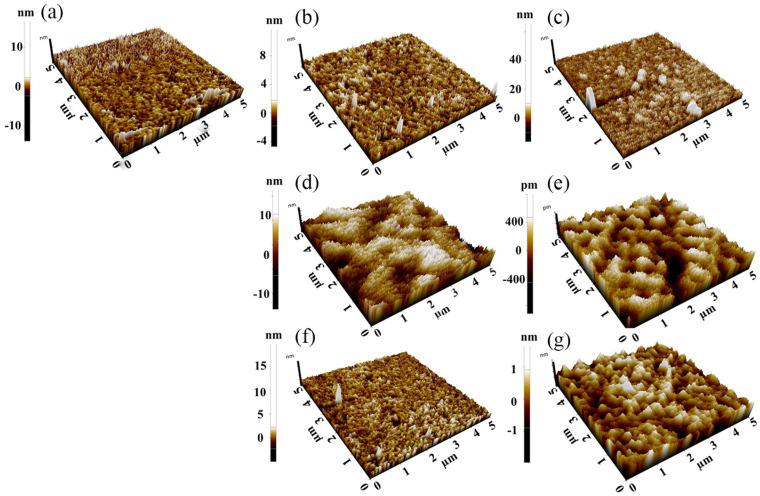
Tapping-mode AFM topography images (5 × 5 µm^2^) of (**a**) bare ITO, (**b**) CH_3_SAM 10 min, (**c**) CH_3_SAM 60 min, (**d**) F_3_SAM 10 min, (**e**) F_3_SAM 60 min, (**f**) NH_2_SAM 10 min, and (**g**) NH_2_SAM 60 min.

Tapping-mode AFM was utilised to determine the surface topography of naked and SAM-modified ITO substrates, as shown in Figure 3. The roughness characteristics collected from each image include arithmetic mean roughness (R_a_), RMS roughness (R_q_), peak-to-valley height (R_pv_), average maximum height (R_z_), skewness (R_sk_), and kurtosis.

Bare ITO has a R_a_ of 0.78 nm and R_pv_ of 31.02 nm. The negative skewness (R_sk_ = −0.83) and high kurtosis (R_ku_ = 26.45) indicate a surface with occasional deep troughs and abrupt peaks, similar to the grain boundary structure of polished ITO glass.

F_3_SAM provided the smoothest surfaces across all samples within 10 min. R_a_ decreased to 0.203 nm and R_pv_ to 2.011 nm, representing 74% and 94% reductions from bare ITO, respectively. At 60 min, the measurements were practically comparable (R_a_ = 0.191 nm, R_pv_ = 1.808 nm), indicating that more vapour exposure did not affect the surface. Both F_3_SAM samples displayed positive skewness and kurtosis values near to 3, indicating a flat Gaussian-like height distribution with modest, isolated protrusions. This behaviour is consistent with self-limiting monolayer chemisorption, in which all available surface hydroxyl sites are occupied within the first few min after deposition, and no further reaction takes place.

CH_3_SAM exhibited a time-dependent reaction in the opposite direction. After 10 min, R_a_ was lowered to 0.681 nm and R_pv_ to 17.615 nm, indicating partial coverage that smoothed some of the original ITO roughness. By 60 min, the surface became much rougher than bare ITO, with R_a_ reaching 3.598 nm and R_pv_ 85.01 nm. The skewness decreased to −2.849, indicating the presence of huge projecting islands over a relatively flat baseline. The high kurtosis (R_ku_ = 26.622) supports the extreme, non-Gaussian height distribution. This profile is typical of inter-silane Si-O-Si condensation after monolayer saturation, in which physisorbed silane molecules combine with previously deposited molecules rather than surface hydroxyl groups, resulting in three-dimensional aggregates.

After 10 min of NH_2_SAM, the roughness values were similar to those of bare ITO (R_a_ = 0.85 nm, R_pv_ = 25.98 nm), with a greater negative skewness (R_sk_ = −1.57) and high kurtosis (R_ku_ = 27.904), indicating a disordered partial monolayer. After 60 min, the surface became smoother (R_a_ = 0.362 nm, R_pv_ = 3.928 nm), with a positive skewness (R_sk_ = +0.32) and near-Gaussian kurtosis (R_ku_ = 3.495), showing that longer deposition facilitated re-organisation into a more compact and uniform monolayer. In contrast to CH_3_SAM, prolonged deposition period had a negative impact on morphology. In summary, F_3_SAM creates a smooth, self-limiting monolayer fast and consistently. CH_3_SAM is particularly sensitive to deposition time and deteriorates after 10 min. 

### 3.4. OLED Electroluminescence Performance

**Figure 4:**
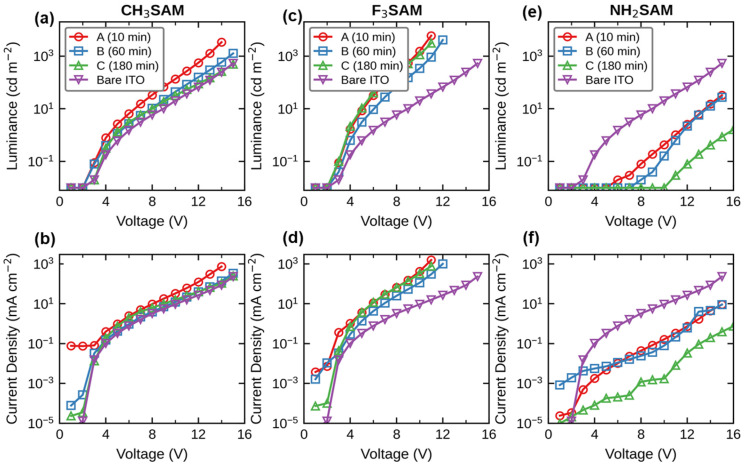
Luminance–voltage (**top**) and current density–voltage (**bottom**) characteristics of (**a**,**b**) CH_3_SAM, (**c**,**d**) F_3_SAM, and (**e**,**f**) NH_2_SAM OLED devices. Bare ITO reference is shown in each panel. Device labels A, B, and C correspond to 10, 60, and 180 min deposition.

**Figure 5:**
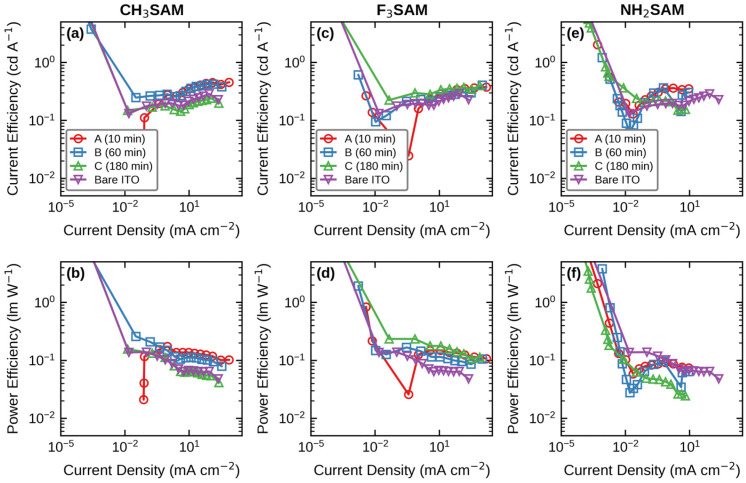
Current efficiency (**top**) and power efficiency (**bottom**) as a function of current density for (**a**,**b**) CH_3_SAM, (**c**,**d**) F_3_SAM, and (**e**,**f**) NH_2_SAM OLED devices. Bare ITO reference is shown in each panel.

Figure 4 and Figure 5 show the electroluminescence characteristics for all devices. The three SAM chemistries yielded dramatically diverse results, ranging from a tenfold increase in luminance over bare ITO to near-complete suppression of light emission.

F_3_SAM devices provided the maximum luminescence across all deposition times. The F_3_SAM-A achieved a maximum luminance of 6000 cd m^−2^ at 11 V, compared to 539 cd m^−2^ for bare ITO, while keeping the same turn-on voltage of 2–3 V. The L–V curves indicate that all three F_3_SAM devices started on rapidly at low voltage and increased significantly, with F_3_SAM-B reaching 4080 cd m^−2^ and F_3_SAM-C reaching 3083 cd m^−2^. The J–V curves show that F_3_SAM-A and F_3_SAM-B had much greater current densities than bare ITO at all applied voltages, indicating improved hole injection at the ITO/NPB interface.

Despite a gradual decrease in maximum luminance from A to C, current efficiency remained nearly constant across all three deposition times (0.37–0.40 cd A^−1^) and power efficiency was consistently around 0.11 lm W^−1^, indicating that charge balance within the emitting zone was well maintained regardless of deposition duration.

CH_3_SAM devices outperformed bare ITO, however the degree of improvement depended heavily on deposition time. CH_3_SAM-A had a maximum luminescence of 3374 cd m^−2^ and the highest current efficiency among all samples (0.45 cd A^−1^). CH_3_SAM-B produced 1263 cd m^−2^, while CH_3_SAM-C produced 490 cd m^−2^. Both luminance and efficiency decreased monotonically throughout deposition time. The J–V curves directly show this pattern, as current density at a given voltage decreases from A to C, which is consistent with the increasing surface roughening described in Section 3.3. The three CH_3_SAM devices maintained the same turn-on voltage as bare ITO (2–3 V), showing that the methyl SAM did not significantly affect the hole injection barrier. NH_2_SAM devices demonstrated the opposite behaviour. Turn-on voltage gradually increased with deposition time (5–6 V for NH_2_SAM-A, 7–8 V for NH_2_SAM-B, and 10–11 V for NH_2_SAM-C). In the L–V graphs, bare ITO significantly outperformed all three NH_2_SAM variants. Maximum luminance decreased to 32.40, 27.53, and 9.96 cd m^−2^ for A, B, and C, respectively. The J–V graphs demonstrate that current density was significantly reduced at low to intermediate voltages, with NH_2_SAM-C displaying almost no current below 10 V. Despite this, maximum current efficiency for NH_2_SAM devices (0.30–0.45 cd A^−1^) remained equivalent to or slightly above bare ITO (0.22 cd A^−1^). This suggests that once holes cross the injection barrier, recombination within the Alq_3_ layer occurs normally. 

## 4. Discussion

### 4.1. Effect of Terminal Group on Work Function and Hole Injection

Although the CF_3_ terminal group of F_3_SAM has intrinsically lower surface energy than the –CH_3_ terminal group of CH_3_SAM, F_3_SAM did not consistently yield higher WCA across all deposition conditions, given that the head and spacer groups are identical for all three SAMs [17]. This is attributed to the greater steric bulk of the fluorinated terminal group, which restricts intermolecular packing and reduces grafting density compared to the compact –CH_3_ terminal group, consistent with previously reported fluorinated SAM systems [18,19]. Barriet and Lee have noted that the packing density of fluorinated SAMs is inherently lower than that of their hydrocarbon analogues owing to the larger cross-sectional area of the CF_2_/CF_3_ groups, so the terminal group’s hydrophobicity advantage can be offset when chain ordering is poor [18]. Consequently, CH_3_SAM achieves a denser and more ordered monolayer over extended deposition times, ultimately yielding higher WCA values at 60 min, demonstrating that monolayer packing order can dominate over intrinsic terminal group surface energy in governing surface wettability. Recent investigations of triethoxysilane SAM formation have confirmed that chemical structure strongly governs both formation kinetics and final film morphology, with subtle differences in reaction conditions producing markedly different surface outcomes [20].

The work function trends observed here are consistent with the well-established surface dipole model, in which the net molecular dipole moment of an ordered monolayer shifts the vacuum level at the electrode surface and thereby raises or lowers the effective work function [11]. Electron-withdrawing –CF_3_ groups create a dipole oriented toward the ITO surface, raising the surface potential and work function, while electron-donating –NH_2_ groups reverse this orientation and lower the work function [9,21]. This mechanism has been widely exploited to engineer ITO anodes for advanced device architectures. Sun et al. demonstrated that a composite SAM combining perfluorobenzyl phosphoric acid with perfluoroalkyl carboxylic acids raised the ITO work function and enabled hole injection and transport layer-free TADF-OLEDs with EQEs of up to 22.0%, underscoring the potential of fluorinated SAMs to streamline OLED device stacks [22]. Similarly, phosphonic acid SAMs bearing electron-withdrawing substituents on ITO yielded maximum luminances of approximately 57,300 cd m^−2^ in green phosphorescent OLEDs, far exceeding the performance of conventional PEDOT:PSS interlayers and confirming that precise work-function alignment at the anode interface is a key driver of luminance and efficiency [21].

Bare ITO has a work function of 3.72 eV, significantly below the NPB HOMO (∼5.4 eV), creating a large hole injection barrier. F_3_SAM raised the ITO work function to 4.72 eV at 60 min, reducing the injection barrier to approximately 0.68 eV and yielding a maximum luminance of 6000 cd m^−2^, nearly 11 times that of bare ITO (539 cd m^−2^). This improvement is particularly noteworthy because it was achieved using a single-component gas-phase deposition without any additional hole injection layer. The device performance was stable across all deposition times, consistent with AFM evidence that F_3_SAM self-limits to a monolayer rapidly. A parallel improvement has been reported for fluorinated silane SAM modification of V_2_O_5_ hole injection layers, where an analogous F8SAM treatment produced a five-fold increase in maximum luminance, confirming that fluorinated silane SAMs are broadly effective for reducing hole injection barriers in organic light-emitting devices [23].

NH_2_SAM lowered the ITO work function below that of bare ITO (3.42 eV at 10 min), deepening the hole injection barrier progressively with deposition time. The turn-on voltage increased from 5–6 V at 10 min to 10–11 V at 180 min as surface coverage grew, while current efficiency remained near 0.30–0.45 cd A^−1^, indicating that charge recombination efficiency within the Alq_3_ emitter was unaffected and that hole supply was the sole limiting factor. This decoupling of carrier injection efficiency from recombination quantum yield is consistent with findings for amine-functionalized ITO surfaces in the literature, where the reduction in work function selectively suppresses hole current without disturbing electroluminescence quantum yield [9]. CH_3_SAM, with its near-zero net dipole, produced a moderate work function increase (3.97 eV at 10 min, 4.62 eV at 60 min) that primarily passivated surface trap states and hydroxyl groups on ITO, consistent with earlier reports attributing performance gains with alkyl-terminated SAMs to suppression of leakage pathways at the anode interface rather than to direct work-function matching [8]. The resulting improvement in hole injection efficiency at 10 min deposition (luminance 3374 cd m^−2^, current efficiency 0.45 cd A^−1^) exceeded that of the F_3_SAM series in terms of efficiency alone, suggesting that trap passivation can be as critical as barrier lowering when the trap density on the ITO surface is high.

### 4.2. Influence of Deposition Time on Surface Morphology and Device Performance

The AFM measurements demonstrate that deposition time affects the three SAMs in very different ways, and the device results are directly related to these morphological variances. F_3_SAM reached its minimum roughness after 10 min (R_a_ = 0.203 nm) and changed negligibly at 60 min (R_a_ = 0.191 nm), demonstrating characteristic self-limiting monolayer formation in which chemisorption of silane head groups to ITO hydroxyl sites reaches saturation before multilayer condensation can proceed. This self-limiting behaviour, attributed to the combined steric shielding from –CF_3_ groups and restricted chain mobility under gas-phase conditions, produced stable and reproducible OLED performance across all three deposition durations (0.37–0.40 cd A^−1^, 0.11 lm W^−1^). Recent mechanistic investigations of triethoxysilane SAM formation have shown that the chemical structure of the silane precursor critically determines whether self-limiting monolayer growth or progressive multilayer condensation occurs: under dry gas-phase conditions, moisture-catalysed hydrolysis and inter-chain Si–O–Si condensation are suppressed, favouring chemisorption-limited, conformal monolayer formation [20]. The gas-phase deposition strategy employed in this work therefore provides an intrinsic kinetic advantage for F_3_SAM relative to solution-phase methods, where dissolved water and physisorbed surface moisture can accelerate cross-condensation and promote island growth.

CH_3_SAM exhibited a time-dependent behaviour in the opposite direction. By 60 min, roughness had significantly increased (R_a_ = 3.598 nm, R_pv_ = 85.01 nm) with highly negative skewness, indicating islanded multilayer development from continuing intra-silane condensation beyond monolayer saturation. These surface aggregates cause local electric field inhomogeneities, increase leakage current, and introduce quenching sites at the organic interface. The device data show luminance decreasing from 3374 cd m^−2^ at 10 min to 490 cd m^−2^ at 180 min, with a corresponding decrease in current efficiency. For CH_3_SAM, 10 min is near-optimal, and extended deposition consistently degrades performance. This behaviour mirrors observations from solution-phase silane SAM studies, where over-deposition leads to island-like multilayer aggregates that disrupt the surface morphology and the quality of subsequently deposited organic layers [8]. The elevated current density observed in the 10 min device below 3 V is attributed to incomplete monolayer coverage at this deposition stage, where residual pinholes establish direct ITO–organic contact pathways that generate ohmic leakage current through trap-assisted tunneling mechanisms, consistent with the intermediate WCA values obtained for CH_3_SAM at 10 min. As deposition time increases to 60 and 180 min, the progressively denser monolayer eliminates these pinhole-mediated leakage pathways, reducing the low-voltage current by approximately four orders of magnitude.

NH_2_SAM revealed the opposite morphological trend—the surface became smoother with longer deposition, with R_a_ lowering from 0.85 to 0.362 nm between 10 and 60 min, and skewness shifting from negative to positive. This behaviour suggests gradual molecular reorganisation into a more compact, upright monolayer, consistent with the known slow chemisorption kinetics of aminopropyl silanes on oxide surfaces, where hydrogen bonding between amine groups and surface silanols promotes initial physisorption followed by slow covalent crosslinking [20]. Despite improved surface order, device performance deteriorated with deposition time because the expanding hole injection barrier caused by increased NH_2_SAM coverage outweighed any benefit from enhanced surface uniformity. Taken together, the three SAM systems demonstrate that morphological evolution and electrical performance are inseparably linked: optimum device performance requires simultaneous control of surface coverage, molecular orientation, and terminal group chemistry.

### 4.3. Gas-Phase Deposition Strategy and Comparison with Literature

The results of this study carry several implications for the design of gas-phase SAM processes for OLED anode engineering. Among the three SAMs studied, F_3_SAM exhibited the most favourable combination of rapid monolayer saturation, upward work function shift, surface smoothening, and deposition-time-independent OLED performance—properties that collectively make it the most suitable candidate for a gas-phase anode treatment. The self-limiting growth behaviour of F_3_SAM under gas-phase conditions represents a process-engineering advantage over solution-phase deposition, which is susceptible to variable moisture content, solvent residues, and precursor concentration gradients that can lead to non-uniform coverage and multilayer formation. Gas-phase thermal deposition at 160 °C, as employed here, minimises these variables and offers a route to solvent-free, vacuum-compatible anode modification compatible with existing OLED manufacturing infrastructure.

Comparing the OLED metrics of the present gas-phase SAM devices with published values places these results in broader context. The F_3_SAM device yielded a maximum luminance of 6000 cd m^−2^ and a current efficiency of 0.37–0.40 cd A^−1^ using a simple fluorescent Alq_3_ emitter, surpassing the performance of bare ITO by nearly one order of magnitude and comparing favourably with solution-phase silane SAM treatments on ITO in similar device architectures [8,9]. Higher absolute luminances and external quantum efficiencies have been reported for more complex architectures employing phosphorescent or TADF emitters in combination with engineered SAM interlayers [21,22,24], reflecting the additional efficiency gains available from triplet harvesting. Enhancing the hole transport layer through post-deposition SAM annealing has also been demonstrated to improve both vertical molecular orientation and hole mobility, delivering maximum luminous intensities exceeding 32,000 cd m^−2^ in fluorescent OLED architectures [24]. The comparatively modest absolute efficiencies of the present devices therefore arise from the choice of emitter rather than from any fundamental limitation of the gas-phase SAM anode treatment. These results collectively demonstrate that terminal group selection and deposition time control are the primary design levers for gas-phase organosilane SAMs on ITO, and that fluorine-terminated silanes offer the most robust performance benefit among commonly available silane coupling agents.

## 5. Conclusions

This study investigated how the gas-phase deposition of three silane SAMs affects ITO anode characteristics and how these changes affect OLED performance. The results suggest that terminal group electronegativity and deposition time are the most important parameters. F_3_SAM was the most successful treatment. After 10 min, it increased the ITO work function to 4.47 eV, reduced surface roughness to R_a_ = 0.203 nm, and achieved a maximum luminance of 6000 cd m^−2^, which is eleven times higher than bare ITO. Performance was consistent throughout all three deposition times, indicating that F_3_SAM monolayer production is self-limiting. After 10 min of CH_3_SAM, surface trap passivation resulted in a useful improvement of 3374 cd m^−2^. However, the roughness increased dramatically as multilayer aggregates developed. NH_2_SAM reduced the ITO work function and created a hole injection barrier that worsened with deposition time, rendering it unsuitable as an anode interlayer in traditional NPB-based OLEDs. Overall, gas-phase F_3_SAM deposition for 10 min at 150 °C provides a straightforward and vacuum-compatible solution to dramatically improve ITO anode performance. This method requires no wet-chemistry stages and generates stable results despite deposition time variation.

## Data Availability

The data presented in this study are available upon reasonable request from the corresponding author.
